# Recurrent Sigmoid Volvulus in a Young Female With Ulcerative Colitis: A Case Report and Review of the Literature

**DOI:** 10.7759/cureus.107089

**Published:** 2026-04-15

**Authors:** Hung-Ming Huang, Yu-Ru Lai, Yu-Chun Yeh, Cheng-Wei Huang

**Affiliations:** 1 Education, China Medical University Hospital, Taichung, TWN; 2 Surgery, China Medical University Hospital, Taichung, TWN

**Keywords:** inflammatory bowel disease, recurrent large bowel obstruction, robotic colorectal surgery, sigmoid volvulus, ulcerative colitis (uc)

## Abstract

Sigmoid volvulus (SV) is extremely rare in young patients with ulcerative colitis (UC). We report a 20-year-old female with UC presenting with recurrent SV without traditional risk factors. Computed tomography (CT) confirmed the diagnosis with a "whirl sign." The patient underwent definitive robotic-assisted anterior resection with inferior mesenteric vessel preservation.

Notably, the site of torsion corresponded to the segment most severely affected by UC, suggesting a localized inflammatory mechanism contributing to volvulus formation.

This case highlights a potential pathophysiological link between chronic inflammation and colonic torsion, possibly involving neuroinflammatory changes. Clinicians should consider recurrent SV in young UC patients as a potential indicator of segmental motility dysfunction and evaluate the need for definitive surgical management.

## Introduction

Sigmoid volvulus (SV) is the most common subtype of colonic volvulus and an important cause of large-bowel obstruction. It occurs when a redundant sigmoid colon twists around its mesenteric axis, resulting in closed-loop obstruction with a risk of bowel ischemia, gangrene, perforation, and sepsis if not promptly treated. Epidemiologically, SV demonstrates marked geographic variation and typically affects older adults in Western populations, whereas it is more frequently encountered in younger patients in endemic regions within the so-called “volvulus belt,” including Africa, South America, Russia, Eastern Europe, the Middle East, India, and Brazil. From a pathophysiologic perspective, SV is strongly associated with anatomical predispositions, particularly an elongated and mobile sigmoid colon or dolichosigmoid and a narrow mesenteric base, which provide the mechanical basis for torsion. Additional risk factors include chronic constipation, advanced age, neuropsychiatric disease, and colonic dysmotility [1-4].

Ulcerative colitis (UC) is a chronic inflammatory disease of the colon characterized by relapsing and remitting mucosal inflammation that typically begins in the rectum and extends proximally in a continuous pattern. It commonly presents with diarrhea and rectal bleeding and represents a major form of inflammatory bowel disease. Its pathogenesis involves complex interactions among genetic susceptibility, environmental exposures, and abnormal immune responses to the intestinal microbiome. Clinically, UC follows a relapsing-remitting course and is associated with substantial morbidity, including an increased long-term risk of colorectal cancer. Although UC primarily affects the colonic mucosa, it may also lead to structural and functional alterations of the bowel, which in rare cases could contribute to unusual complications [5]. 

The coexistence of SV and UC is extremely rare, with only three cases reported in the literature, including one pediatric case of SV and two adult cases involving sigmoid and cecal volvulus, respectively. Although the pathophysiological association remains incompletely understood, chronic inflammation likely plays a central role in predisposing patients with UC to volvulus formation. Chronic inflammation may result in tissue remodeling, including muscular weakening, subepithelial fibrosis, impaired peristalsis, and altered colonic motility. These changes, together with colonic elongation, redundancy, and acquired megacolon, may create a mobile bowel segment with a relatively narrow mesenteric base that is more susceptible to torsion. In addition, fecal stasis and constipation may further increase the risk of volvulus in susceptible patients [6,7].

Because SV may present similarly to other acute colonic complications of UC, particularly toxic megacolon, prompt differentiation is critical to guide appropriate intervention. Herein, we report a rare case of recurrent SV in a young woman with long-standing UC, highlighting the diagnostic challenge of this unusual association and the rationale for definitive surgical management.

## Case presentation

A 20-year-old female with a six-year history of UC treated with mesalazine (Mayo score: 6) presented to the emergency department with severe abdominal distension for one day. Two months prior, she had experienced a similar episode of SV that was temporally decompressed by endoscopic detorsion. 

**Figure 1:**
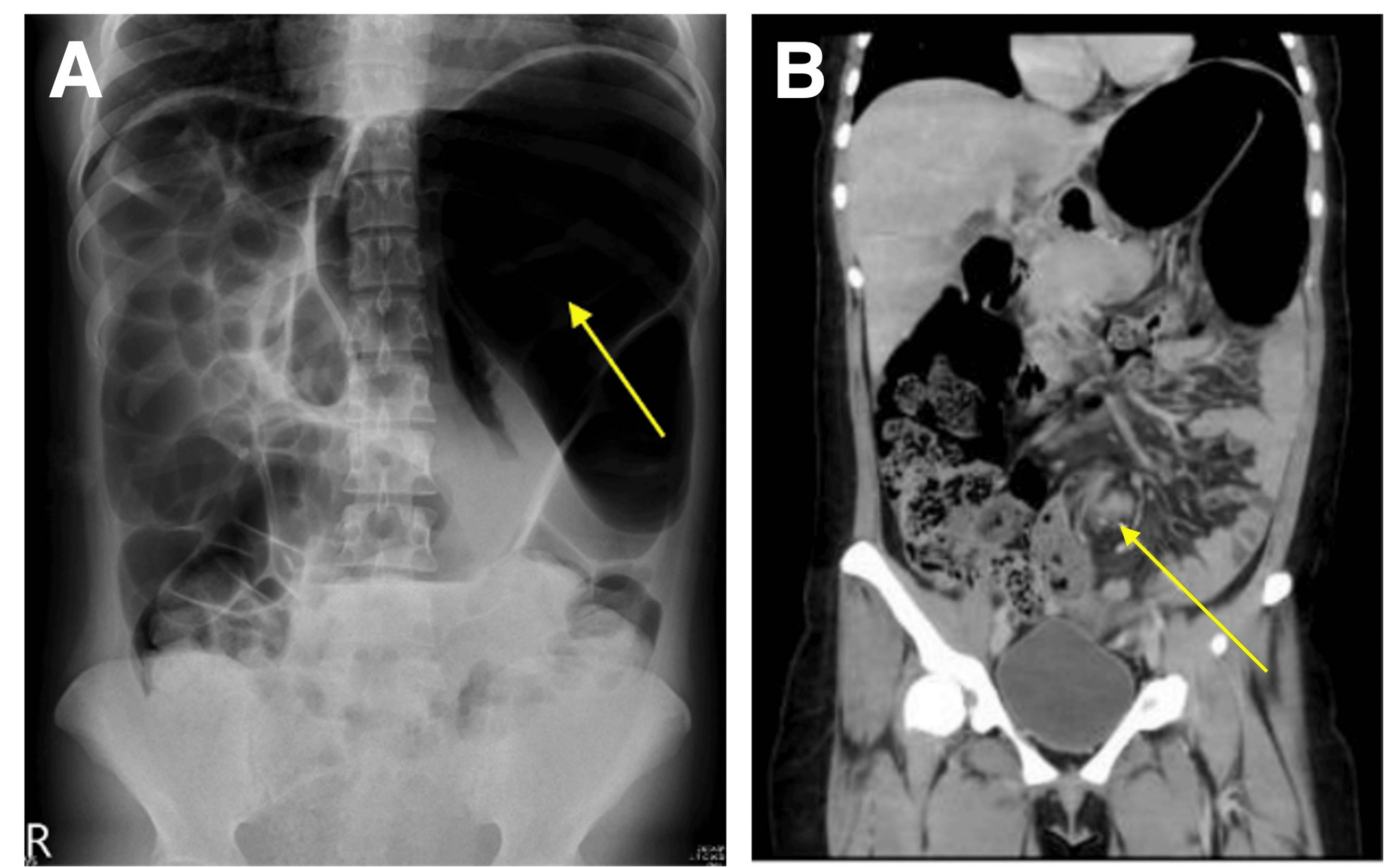
Radiographic findings of the sigmoid volvulus A: abdominal radiograph demonstrating the characteristic "reversed coffee bean sign" (yellow arrow) with a marked absence of rectal gas; B: coronal view of abdominal computed tomography revealing a dilated sigmoid colon with the pathognomonic "whirl sign" (yellow arrow), indicating torsion of the mesentery.

Physical examination revealed a distended abdomen with hypoactive bowel sounds and lower abdominal tenderness. Laboratory tests showed leukocytosis with left shift and elevated C-reactive protein with 3.09 mg/dL (reference range <1.0 mg/dL). Abdominal radiography demonstrated the classic "reversed coffee bean sign" and absence of rectal gas (Figure 1A). Computed tomography (CT) confirmed a markedly dilated sigmoid colon with the characteristic "whirl sign" (Figure 1B). Although the plain abdominal radiograph suggested a SV, an abdominal CT scan was arranged to rule out complications such as bowel ischemia or perforation, to assess the current severity and extent of the underlying UC, and to facilitate precise preoperative planning.

**Figure 2:**
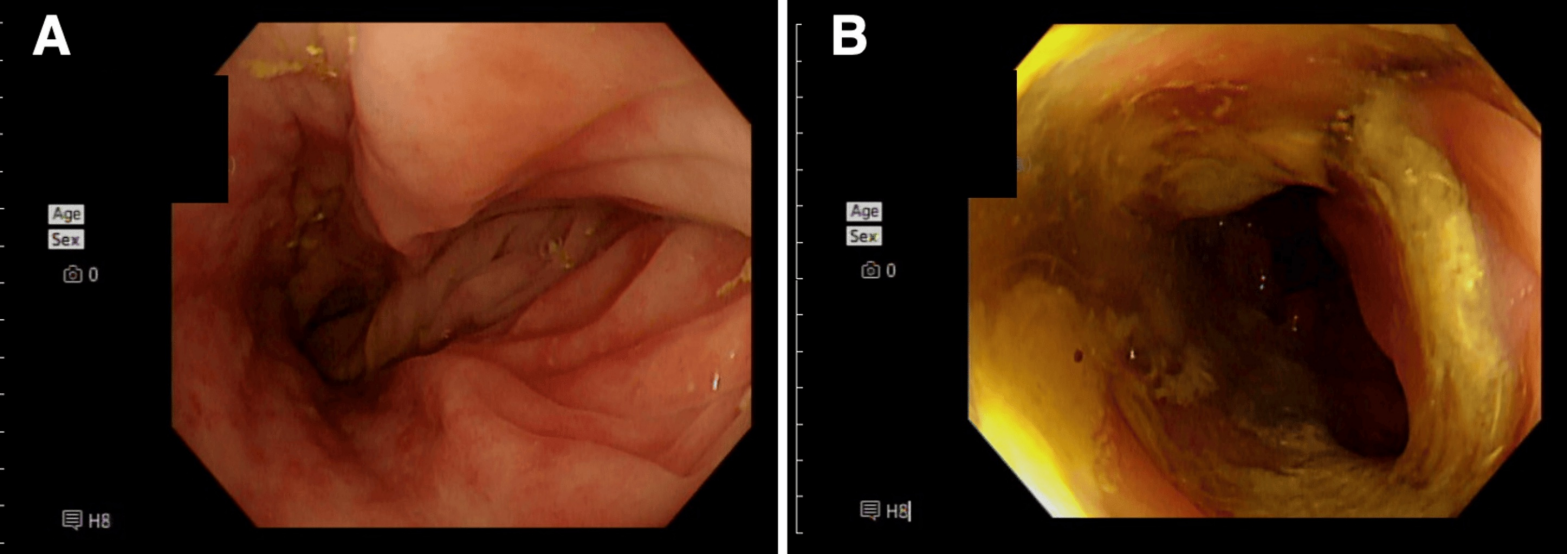
Endoscopic findings of the sigmoid colon A: representative view showing diffuse mucosal inflammation with loss of normal vascular pattern and patchy friability; B: endoscopic appearance consistent with severe active ulcerative colitis characterized by deep ulcerations, spontaneous bleeding, and prominent, thick fibrinous exudates overlaying the severely inflamed mucosa.

Emergent colonoscopic detorsion acutely relieved the obstruction. Notably, the torsion site corresponded to the colonic segment most severely affected by UC inflammation (Figure 2A, 2B).

**Figure 3:**
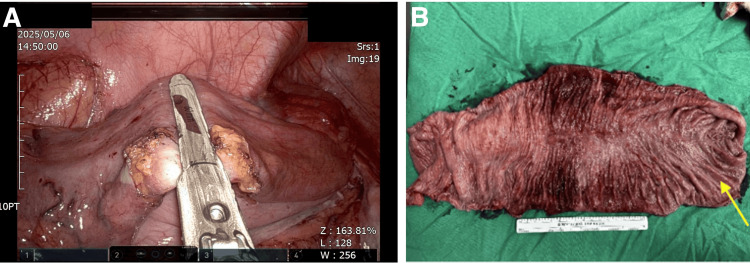
Intraoperative and pathological findings A: intraoperative view showing the completed colocolic anastomosis performed via the robotic-assisted approach; B: gross specimen of the resected sigmoid colon (yellow arrow indicates the distal end). The mucosa exhibits extensive ulceration and inflammatory changes consistent with active ulcerative colitis. Notably, the segment with the most severe inflammation corresponds to the site of torsion, with no gross evidence of malignancy.

Due to the recurrence of SV, the patient consented to definitive surgical management. A robotic-assisted anterior resection was performed one week after the colonoscopic intervention to ensure the patient's condition was stable. Intraoperatively, the inferior mesenteric vessels were carefully preserved to maintain optimal colonic perfusion. Selective ligation of sigmoid arterial branches was performed, and lymph node dissection was deemed unnecessary in this case. A colocolic anastomosis was created 25 cm from the anal verge (Figure 3A). Pathological examination of the resected specimen confirmed features consistent with UC. Microscopic evaluation revealed diffuse mucosal hemorrhage, moderate lymphoplasmacytic infiltration, and mild neutrophilia with crypt abscesses. Additional chronic mucosal changes included shortened villi, decreased goblet cells, and several lymphoid follicles with germinal centers. The bilateral resection margins were viable with focal serosal fibrosis, and there was no evidence of malignancy (Figure 3B).

The postoperative course was uneventful. The patient was discharged on postoperative day five after inflammatory markers (WBC and high-sensitivity C-reactive protein (hsCRP)) normalized (white blood cell count reference range: 4,000-10,000 /μL). At the six-month follow-up, she remained asymptomatic with well-controlled UC.

## Discussion

SV is a well-known cause of large-bowel obstruction in the elderly but remains rare in pediatric and young adult populations [4]. Typical predisposing factors for SV include neuromuscular disorders, Hirschsprung disease, redundant sigmoid colon, and chronic constipation [6,8-11]. However, our patient presented without these typical risk factors, having only a six-year history of UC.

The coexistence of SV and UC is extremely rare. To our knowledge, only one pediatric case of obstructing SV in a patient with UC has been reported (Table 1) [6]. In addition, one adult case has been described with simultaneous transverse and sigmoid colon volvulus presenting as non-toxic megacolon (Table 1) [7]. 

**Table 1:** Summary of reported cases of colonic volvulus in patients with ulcerative colitis This table compares the demographic characteristics, clinical features, management strategies, and outcomes of previously reported cases with the present case. UC: ulcerative colitis; F: female

Author (Year)	Age / Sex	UC Duration	Volvulus Site	Management	Outcome
Mittal A et al. (2023) [6]	16 / F	Not stated	Sigmoid	Endoscopic detorsion	Recovered; no recurrence at 12 months
Katsanos K et al. (2009) [7]	83 / F	Five years	Transverse & Sigmoid	Open subtotal colectomy	Recovered
Present Case (2026)	20 / F	Six years	Sigmoid	Robotic-assisted anterior resection	Recovered; no recurrence at six months

Compared with previously reported cases, our patient is among the youngest adults with recurrent SV, and notably, the torsion occurred at the segment most severely affected by UC. These observations suggest that chronic inflammatory bowel disease may contribute to altered colonic anatomy and motility, thereby serving as a potential predisposing factor for SV.

The pathophysiological relationship between UC and SV remains unclear. Possible mechanisms include chronic mucosal and submucosal inflammation leading to fibrosis and structural distortion of the colon, which shortens or stiffens the intestine and leads to motility disorder [12,13] and pre-existing anatomical variations such as a redundant sigmoid loop. Mentioned in the report by Mittal A et al., the stiff, shortened segment may behave as a relative point of fixation, while the redundant sigmoid loop more readily rotates around the mesenteric axis [6]. Rather than generalized dysmotility, this case suggests that localized severe inflammation may create a focal pivot point, predisposing the affected segment to torsion. 

We hypothesize that chronic inflammation may promote local structural and motility changes that predispose the affected segment to torsion. Prior studies have described neuromuscular and myenteric plexus abnormalities in UC [14]; however, our case did not include a dedicated full-thickness histopathological or immunohistochemical evaluation of these changes. Therefore, the proposed neurogenic mechanism remains speculative. In our case, the main finding supporting a pathogenic link is the spatial correlation between the site of torsion and the colonic segment most severely affected by UC.

In the present case, the recurrence of SV in a young UC female without traditional risk factors highlights the need for heightened clinical suspicion despite its rarity. Diagnosis can be challenging as SV symptoms, abdominal distension, and pain overlap with UC complications like toxic megacolon or severe flares. Therefore, radiographic findings such as the “coffee bean sign” and CT evidence of a “whirl sign” are crucial for timely and accurate diagnosis [4]. Early identification is essential to prevent life-threatening ischemia, necrosis, or perforation.

Initial endoscopic detorsion effectively relieves acute obstruction but carries a high recurrence risk. This high recurrence occurs because the redundant sigmoid colon and elongated mesentery remain unresected, allowing for repeated torsion. Reported long-term recurrence rates reach 43-90%, with patients averaging 4.4 volvulus attacks [15]. Consequently, definitive surgical intervention is recommended, particularly for young patients or recurrent cases. The 2023 World Society of Emergency Surgery (WSES) consensus recommends elective sigmoidectomy after recurrent episodes, even in young patients, to prevent ischemic complications [4]. Accordingly, we performed a robotic-assisted anterior resection. By preserving the inferior mesenteric vessels, we ensured optimal perfusion and achieved rapid recovery while preventing future recurrence. 

## Conclusions

This case highlights a rare association between UC and SV in a young patient without traditional risk factors. The correspondence between the site of torsion and the most severely inflamed colonic segment raises the possibility that localized inflammation may have contributed to torsion, although this relationship remains speculative. Recurrent SV in young patients with UC should prompt consideration of early definitive surgical management to prevent further episodes and complications.
